# The availability, prices and affordability of essential medicines in Malawi: A cross-sectional study

**DOI:** 10.1371/journal.pone.0212125

**Published:** 2019-02-12

**Authors:** Felix Khuluza, Christine Haefele-Abah

**Affiliations:** 1 Pharmacy Department, College of Medicine, University of Malawi, Blantyre, Malawi; 2 Pharmaceutical Institute, Eberhard-Karls-University Tuebingen, Tuebingen, Germany; Ministry of Health and Sports, MYANMAR

## Abstract

**Introduction:**

The Malawian government recently introduced cost-covering consultation fees for self-referral patients in tertiary public hospitals. Previously, patients received medicines free of charge in government-owned health facilities, but must pay elsewhere. Before the government implements a payment policy in other areas of health care, it is important to investigate the prices, affordability and availability of essential medicines in Malawi.

**Methods:**

Data on availability and prices of 50 essential medicines were collected in 44 health facilities in two major cities and two districts. These included 12 public facilities, 11 facilities of the Christian Health Association of Malawi (CHAM), nine retail pharmacies, eight wholesalers and four private clinics/hospitals. Price, availability and affordability were assessed based on the methodology developed by the World Health Organization and Health Action International, which compares local prices to international reference prices.

**Results and discussion:**

The overall availability of medicines was 48.5% in public facilities, 71.1% in retail pharmacies, 62.9% in CHAM facilities and 57.5% in private clinics. The availability of essential medicines varied from 0% for ethosuximide to 100% for amoxicillin and cotrimoxazole tablets. Antibiotic formulations for adults were widely available, in contrast to the low availability of pediatric formulations. Several medicines for non-communicable diseases like sodium valproate, phenytoin, paraldehyde, captopril and simvastatin showed poor availability and affordability. The overall median price ratio compared to the international reference price was 1.11 for wholesalers, 2.54 in CHAM facilities, 2.70 in retail pharmacies, and 4.01 in private clinics, which is low compared to other countries. But nevertheless, for 18 out of 32 medicines assessed, the cost of one course exceeded the statutory minimum daily wage, making them unaffordable to a majority of the population. Therefore, continued provision of free public health care is still of critical importance for the foreseeable future until other financing mechanisms have been explored.

## Introduction

In Malawi, health care is provided by both the public and the private sector. Up to 60% of the health care services are provided by the public facilities, while 37% are provided by Christian Health Association of Malawi (CHAM) and 3% by the private sector [1, 2]. Health care services in the public sector are provided free of charge, whereas in CHAM and private sector facilities, patients pay for their medicines and services. Because of the rapid population growth in Malawi, the financing of the free public health care services represents a major challenge for the Malawian government. At the current rate of population growth of 3.26%, the population of Malawi is projected to reach 26,585,000 by 2030 compared to the current population of 18,850,000 [3, 4].

Continued free public health care implies increased funding through taxation or through international partners. However, due to the revelations of the massive plunder of public resources in Malawi in 2013 [5], the majority of international partners have switched from direct to indirect aid, which further complicates health service financing and delivery in Malawi. Malawi must look for other sources of income for the delivery of health services delivery. Therefore, cost-recovery measures are being considered in the public sector including the health sector.

In 2014, the government of Malawi introduced a by-pass fee of 3.4 US $ in all central (tertiary) hospitals for patients arriving without a referral letter from a secondary (district) hospital. The reasons for this were two-fold: to reduce crowding at tertiary facilities and over-reliance on tertiary care as well as raising additional funds for the tertiary hospitals. This created anxiety in people living in cities that do not have secondary health facilities. At the same time, the by-pass fee is being considered as the first step in cost-recovery (or co-pay) with the likelihood of further charges for medication in tertiary hospitals before the system is introduced in other public facilities [6] as envisioned by the Malawi National Medicine Policy (MNMP) of 2015. The section on ‘Medicine Financing’ of the Policy states: “*The Ministry of Health (MoH) shall provide to patients free of charge at the point of delivery medicines funded by Government of Malawi*. *However*, *the MoH shall explore mechanisms for co-payment after undertaking thorough consultations with relevant stakeholders*.” [7]

If medicines are not available in public or CHAM facilities, patients are referred to private pharmacies to buy the necessary medicines, paying for themselves without re-imbursement except for the few people who are covered by private medical insurance. Prescription only medicines (POM) may commonly be purchased without a prescription in private pharmacies in Malawi [8, 9]. Despite the many challenges facing the financing of the Malawian health care system and possible attempts to switch to the co-pay system, there are limited data on prices, affordability and availability of essential medicines in Malawi to inform the policy makers on the appropriate pricing as well as the suitability of the co-pay system for Malawians. So studies are needed to generate data for the process of making decisions about prices and affordability and create a system that is responsive to the mutual needs of the society and the health care system.

Therefore, the study aimed to determine the availability, prices and affordability of essential medicines in Malawi, especially focusing on tracer medicines. Tracer medicines are a special group of medicines, which the Malawian government selected as a way of monitoring medicine availability in the public sector. The availability of tracer medicines in a health facility signifies that basic health care is available to the community, while their unavailability signifies the deterioration of the pharmaceutical supply chain, which may hinder the achievement of the UN Sustainable Development Goal 3 on Health [10]. This study focused on 50 essential medicines and thus expanded the range of products compared to previous studies that focused mainly on antimalarial and antibiotic medicines [11, 12].

## Methodology

The study was a descriptive cross-sectional survey to determine availability, prices and affordability of 50 essential medicines in medicine outlets from all relevant sectors including public health facilities, CHAM health facilities, private clinics, private retail pharmacies and wholesalers. The methodology developed by the World Health Organization (WHO) and Health Action International (HAI) on measuring medicine prices, availability and affordability was applied, where possible, for the development of the study design, data collection and analysis [13].

### Study sites and sampling procedure

The study was conducted in two of the four Malawian cities, Lilongwe and Blantyre, and in two districts (Mchinji and Salima) where only CHAM facilities were sampled to compensate for the lower number of CHAM facilities in the cities. The cities were selected with regard to the probability of finding and locating enough private retail pharmacies and clinics. WHO/HAI methodology recommends the sampling of at least five randomly selected medicine outlets per sector. During the time of the study, there were enough private pharmacies in Lilongwe and Blantyre to allow for random sampling (29 pharmacies in Lilongwe, and 26 in Blantyre out of a total of 68 registered pharmacies in the country in 2017).

Sampling was also conducted in public, CHAM and private hospitals/clinics as well as with registered pharmaceutical wholesalers. Lists of public facilities, private clinics/hospitals and CHAM facilities from the two cities were obtained from the Ministry of Health and random selection of facilities was done using STATA 13.1. Lists of registered pharmaceutical wholesalers and retail pharmacies were provided by the country’s Pharmacy Medicines and Poisons Board, where random selection of retail pharmacies was made. At the time of data collection, there were a total of 86 pharmaceutical wholesalers registered in Malawi, 47 in Lilongwe, 32 in Blantyre and seven elsewhere. From these facilities, data were collected from eight wholesalers, four in Blantyre and four in Lilongwe which were randomly selected.

Five sample sites for each category in each of the two selected cities were chosen except for the public facilities, where a central hospital, the district health office and four randomly selected health centres (primary health facilities) were taken to cover the different levels of health care. The CHAM facilities were randomly selected. Some facilities (one retail pharmacy, six private clinics and two wholesalers) declined to provide data on both prices and availability, even though confidentiality of data was assured.

**Fig 1:**
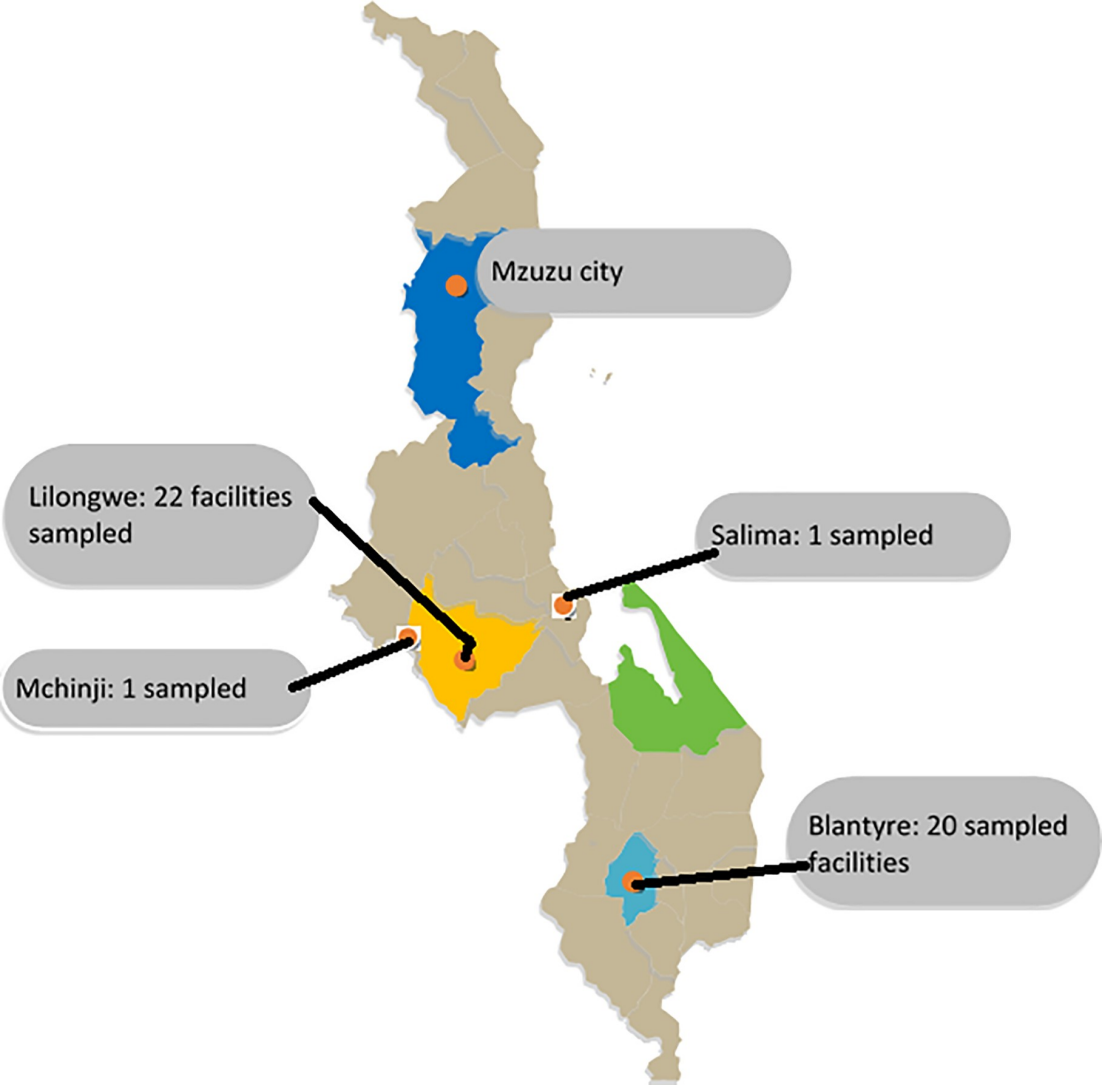
Map of Malawi showing sampled districts. Available from: https://yourfreetemplates.com/free-malawi-map-template/.

This resulted in data from 44 facilities comprising 12 public (government-owned) health facilities, 11 CHAM facilities, nine community/retail pharmacies, eight wholesalers and four private general clinics/hospitals (see Fig 1 of the map of Malawi showing the location of the sampled areas). Price information data for the sole public wholesaler, Central Medical Stores Trust (CMST) was gathered from their website on June 30^th^, 2017, the month in which data were collected. The wholesalers´ data were primarily collected for the pricing analysis. Analysis and presentation of availability data were focused on the level of health facilities, excluding wholesalers´ data.

### Criteria for selection of medicines for the study

Information on availability and prices was collected for 50 different medicines based on the Malawi Essential Medicines List, the “List of Tracer Medicines for Measuring Stockouts” as provided by the Ministry of Health (see S1 Table) and WHO/HAI Global core list of essential medicines [13]. Table 1 lists all medicines included in the study while the S2 Table provides the reasons for inclusion in the study.

**Table 1:** Medicine availability in different types of health facilities in Southern and Central Malawi.

Categori-zation according to MEML 2015^1^	Medicine name (including strength and dosage form)	Source (medicine list)	Public facilities (n = 12)	Private pharmacies (n = 9)	CHAM facilities (n = 11)	Private clinics (n = 4)
HVA	Acyclovir 400mg cap/tab	MEML	8.3%	100.0%	36.4%	25.0%
HVA	Acyclovir 5% cream	MEML	0.0%	100.0%	54.5%	50.0%
HEA	Albendazole 10% suspension	MEML	0.0%	88.9%	9.1%	50.0%
HVA	Amitriptyline 25mg tab	Global	41.7%	88.9%	81.8%	75.0%
HVA	Amoxicillin 250mg cap/tab	Global, MEML, Tracer list	100.0%	100.0%	100.0%	100.0%
HVA	Amoxicillin 25mg/mL suspension	MEML, Tracer list	33.3%	77.8%	81.8%	100.0%
HVA	Benzylpenicillin 5 MU injection	MEML, Tracer list	100.0%	66.7%	100.0%	25.0%
-	Bisoprolol 5mg tab	Global	8.3%	55.6%	0.0%	25.0%
CVB	Captopril 25mg tab	Global, MEML	16.7%	100.0%	36.4%	50.0%
DVA	Carbamazepine 200mg tab	Global, MEML	83.3%	100.0%	63.6%	50.0%
DVA	Ceftriaxone 1 g in vial, powder for injection	Global, MEML	100.0%	88.9%	90.9%	75.0%
CVB	Cimetidine 400mg tab	Global, MEML	41.7%	100.0%	18.2%	25.0%
DVB	Ciprofloxacin 500mg tab	Global, MEML	83.3%	100.0%	100.0%	100.0%
HVA	Cotrimoxazole 480mg tab	MEML, Tracer list	100.0%	100.0%	100.0%	100.0%
H	Cotrimoxazole 48mg/mL suspension	Global, MEML, Tracer list	16.7%	100.0%	63.6%	100.0%
DEA	Diazepam 5mg tab	Global, MEML, Tracer list	33.3%	100.0%	54.5%	50.0%
DVA	Diazepam 5mg/mL injection	MEML, Tracer list	91.7%	22.2%	90.9%	50.0%
DEA	Diclofenac 50mg tab	Global, MEML	83.3%	66.7%	100.0%	100.0%
HVA	Ergometrine maleate 200mg/mL injection	MEML	0.0%	11.1%	9.1%	0.0%
HVA	Erythromycin 250mg tab	MEML	41.7%	77.8%	100.0%	100.0%
CEB	Ethosuximide 250mg cap/tab	MEML	0.0%	0.0%	0.0%	0.0%
DVA	Fluconazole 200mg cap/tab	MEML	50.0%	88.9%	63.6%	50.0%
HVA	Gentamicin 80mg/2mL injection	MEML, Tracer list	100.0%	88.9%	100.0%	100.0%
DEA	Griseofulvin 125mg tab	MEML	41.7%	77.8%	81.8%	25.0%
DVA	Hydrochlorothiazide 25mg tab	MEML	100.0%	88.9%	100.0%	100.0%
DEA	Ibuprofen 200mg cap/tab	MEML	100.0%	100.0%	90.9%	100.0%
DVA	Insulin 100 IU/mL soluble injection, 10 mL	MEML	25.0%	55.6%	36.4%	25.0%
DVA	Insulin zinc 100 IU/mL suspension injection, 10 mL	MEML	25.0%	33.3%	36.4%	25.0%
HVA	Magnesium sulphate 50% injection	MEML	75.0%	0.0%	72.7%	50.0%
HVA	Mebendazole 500mg tab	MEML	0.0%	22.2%	9.1%	25.0%
DVA	Metformin 500mg tab	Global, MEML	41.7%	100.0%	54.5%	100.0%
DEA	Methyldopa 250mg tab	MEML	75.0%	66.7%	54.5%	50.0%
HVA	Metronidazole 200mg tab	MEML, Tracer list	100.0%	88.9%	100.0%	100.0%
DVA	Misoprostol 200mcg tab	MEML	16.7%	77.8%	54.5%	50.0%
DVA	Omeprazole 20mg cap	Global, MEML	58.3%	88.9%	100.0%	100.0%
HVA	Oxytocin 10 IU/mL injection	MEML, Tracer list	83.3%	0.0%	90.9%	50.0%
HVA	Paracetamol 500mg tab	MEML	100.0%	100.0%	90.9%	100.0%
HVA	Paracetamol 24mg/mL suspension	Global, MEML	25.0%	100.0%	81.8%	100.0%
HVA	Paraldehyde injection 10 mL vial	MEML	0.0%	0.0%	9.1%	0.0%
HVA	Phenobarbital sodium 200mg/mL injection	MEML	25.0%	11.1%	54.5%	50.0%
HVA	Phenobarbital sodium 30mg tab	MEML	83.3%	100.0%	100.0%	50.0%
DVA	Phenytoin sodium 50mg/mL injection	MEML	0.0%	0.0%	0.0%	25.0%
DVA	Phenytoin sodium 100mg tab	MEML	50.0%	66.7%	36.4%	0.0%
H	Praziquantel 600mg tab	MEML	66.7%	88.9%	63.6%	25.0%
DVA	Salbutamol inhaler	Global, MEML	0.0%	100.0%	81.8%	100.0%
CVA	Simvastatin 20mg cap/tab	Global, MEML	8.3%	77.8%	9.1%	0.0%
HVA	Sodium chloride 0.9% injection	MEML, Tracer list	67.0%	33.3%	90.9%	50.0%
CVA	Sodium valproate 200mg cap/tab	MEML	25.0%	66.7%	9.1%	50.0%
HVA	Tetracycline 1% eye ointment	MEML, Tracer list	33.3%	88.9%	81.8%	75.0%
HVA	Zinc sulphate 20mg tab	MEML	66.7%	100.0%	100.0%	50.0%

1 = The Malawi Essential Medicines List (MEML) of 2015 specifies the level of health institution at which the medicine is normally permitted for use: H = at health centre, district hospital and central hospital levels; D = at district hospital and central hospital levels only; C = at central hospital level only; N = level of use not specified. The ‘therapeutic priority’ code categorizes medicines based on therapeutic importance of each medicine by the use of: V = vital medicines which are potentially life-saving, of major public health relevance and having significant withdraw side-effects; E = essential medicines which are effective against less severe, but nonetheless significant forms of illness; N = non-essential medicines which are used for minor self-limiting illness and are often of questionable efficacy. The third categorization of ‘procurement system’ has two codes: ‘A’ = medicines required by a large number of patients as such to be routinely procured and stocked by CMST; and ‘B’ = medicines required for a limited number of patients and not routinely stocked by CMST

The Malawi Essential Medicines List (MEML) 2015 [14] categorizes medicines by using a three code system based on ‘level of use’. The ‘level of use’ specifies the health institution where the medicine is normally permitted for use: H = at Health centre, District hospital and Central hospital levels; D = at District hospital and Central hospital levels only; C = at Central hospital level only [12]. The 50 sampled medicines contained examples from each of these three levels of use, as well as medicines not contained in the MEML.

The “List of Tracer Medicines for Measuring Stockouts” was provided by MoH (Chimenya C, MoH, personal communication, 2017) and contained 29 items, as shown in S1 Table. Of these items, 11 medicines were included. The rest of the products were left out because they were either medical/surgical supplies or products provided to Malawian government through donations e.g. antimalarials, and anti-Tuberculosis medicines. Furthermore, all 14 medicines from the WHO/HAI Global core list of medicines were included.

Of special note was the inclusion of medicines for epilepsy as an example of a rather neglected non-communicable disease (NCD) and of maternal health products. The full range of antiepileptics [14] found on the MEML was included as were other medicines targeting NCDs, specifically hypertension and diabetes (hydrochlorothiazide, metformin and insulins). Maternal health medicines were included as a way of assessing efforts to address the high maternal mortality in Malawi. The major maternal medicines used in Malawi and selected for the study were misoprostol tablets, oxytocin injection, ergometrine injection, magnesium sulphate injection and methyldopa.

### Data collection, calculation of medicine availability and prices

The data were collected by two trained teams of two persons each. The development of the questionnaire and the verification of data accurateness were based on the WHO/HAI template and methodology [13] with minor modification as suggested by Marg Ewen of Health Action International (Ewen M., personal communication, January 2017). The modified data collection form has been submitted as S3 Table. The data collectors recorded the medicine availability and price as provided by the responsible personnel, mainly the person in charge of the pharmacy. In the public health facilities, information was collected on availability only, as the public sector provides medicines free of charge [13].

Medicine availability was calculated as the percentage of facilities which had stock of the respective medicine at the time of the visit, irrespective of the amount and pack size available, as recommended by the WHO/HAI methodology. The WHO has set a minimum of 80% as target availability of medicines for both communicable and non-communicable diseases in all countries [15, 16]. This cutoff point has been used in analyzing the availability of medicines for this study.

Medicine prices were recorded in local currency (Malawi Kwacha, MWK). Prices were converted to US $ using the average Reserve Bank of Malawi [17] exchange rate for the month of June 2017 (1 US $ = 725.7662 MWK), the month in which the data were collected. The median price ratio (MPR) for each medicine was calculated by dividing the observed median prices by an international reference price taken from the MSH International Medicine Price Indicator Guide of 2015 [18]. This is usually an ex-works price (EXW) from international wholesalers, not including insurance and freight.

The study aimed to collect prices for originator brands, the most commonly sold generics and the lowest priced generics. However, during data collection, originator brands were not found and for most items, only one product/price was available. Therefore, categorization of the results into the three groups as initially planned was not done. Where two prices/products or more were available, the cheapest item was included in the data analysis. However, the unavailability of lowest priced generics and originator brands, and thus lack of categorization, did not have an impact on the results of the study.

### Calculation of courses of treatment and of medicine affordability

Affordability of medicines was assessed for prevailing diseases in Malawi based on the global burden of diseases [19] and priority research areas for communicable and non-communicable diseases in Malawi [20], considering therapeutic groups of the selected medicines. The diseases that were selected for calculation of affordability of medicines in Malawi were:

- For antibiotics: Upper and lower respiratory diseases, neonatal sepsis, meningitis, urethritis and abnormal vaginal discharge;
- For maternal health: Post-partum hemorrhage (PPH), pre-eclampsia and eclampsia;
- For non-communicable diseases: Asthma, hypertension, stroke, type 1 and 2 diabetes;
- For mental disorders: Seizures, epilepsy, anxiety, and psychosis.

Thus, a subset of 32 medicines of the whole range of medicines studied was included for the calculation of affordability. Malawi Standard Treatment Guidelines (MSTG) 2015 [14], WHO/HAI manual and WHO ATC/DDD Index [21] were used to calculate the amount of tablets/capsules/vials required for one course of treatment or for a monthly treatment in case of chronic conditions. For ceftriaxone and benzylpenicillin injections, a treatment duration of three days (before changing to oral medication) was chosen. As for gentamicin, a single dose of 240mg was used for the calculation of affordability which is a single dose for urethritis and abnormal vaginal discharge in combination with metronidazole 2g and erythromycin 500mg four times daily for seven days. For pediatric formulations, one bottle of 100ml was used as a complete dose. The daily minimum wage of the lowest-paid unskilled worker, as legislated by the Malawian government, was used to measure local affordability and the number of days’ wages needed to purchase a course of treatment (962 MWK = 1.33 US$) [22].

### Ethics approval and consent to participate

The study was approved by the College of Medicine Research and Ethics Committee, COMREC number P.03/17/2131 and by the Pharmacy Medicines and Poisons Board. Respondents to questionnaires were asked for their consent before the commencement of data collection at any facility.

## Results

### Availability of medicines

The overall availability of medicines in various sectors was 48.5% for public, 71.1% for retail pharmacies, 62.9% for CHAM and 57.5% for private clinics. Availability of medicines varied from as low as 0% of ethosuximide tablets in all sectors to 100% of amoxicillin capsules/tablets and cotrimoxazole tablets in all health facilities, as shown in Table 1.

The availability of medicines in the public sector varied across different levels of care. For medicines which were earmarked to be available on all levels (H), the availability was 47% in the primary health centres, 56% in the district and 66% in the central hospitals. However, medicines which were supposed to be found at district level and above found their way to the primary health centres with an availability of 48% at this level and 68% and 82% at the district and central hospital levels respectively. Similarly, some tertiary-level (C) medicines found their way to the lower levels with an availability of 9%, 20% and 60% in the primary health centres, district hospitals and central hospitals respectively. See Table 2.

**Table 2:** Disaggregated data on availability of medicines in public health facilities in Malawi.

Level of use based on MEML 2015	Medicine name (including strength and dosage form)	Overall availability in public health facilities	Public health centres (n = 9)		Public district hospitals (n = 1)		Central hospitals (n = 2)	
H	Aciclovir 400mg cap/tab	8.3%	1/9	11%	0/1	0%	0/2	0%
H	Aciclovir 5% cream	0.0%	0/9	0%	0/1	0%	0/2	0%
H	Albendazole 10% suspension	0.0%	0/9	0%	0/1	0%	0/2	0%
H	Amitriptyline 25mg tab	41.7%	3/9	33%	0/1	0%	2/2	100%
H	Amoxicillin 250mg cap/tab	100.0%	9/9	100%	1/1	100%	2/2	100%
H	Amoxicillin 25mg/mL suspension	33.3%	2/9	22%	0/1	0%	2/2	100%
H	Benzylpenicillin 5 MU injection	100.0%	9/9	100%	1/1	100%	2/2	100%
-	Bisoprolol 5mg tab	8.3%	0/9	0%	0/1	0%	½	50%
C	Captopril 25mg tab	16.7%	1/9	11%	0/1	0%	½	50%
D	Carbamazepine 200mg tab	83.3%	7/9	78%	1/1	100%	2/2	100%
D	Ceftriaxone 1 g in vial, powder for injection	100.0%	9/9	100%	1/1	100%	2/2	100%
C	Cimetidine 400mg tab	41.7%	2/9	22%	1/1	100%	2/2	100%
D	Ciprofloxacin 500mg tab	83.3%	7/9	78%	1/1	100%	2/2	100%
H	Cotrimoxazole 480mg tab	100.0%	9/9	100%	1/1	100%	2/2	100%
H	Cotrimoxazole 48mg/mL suspension	16.7%	0/9	0%	0/1	0%	2/2	100%
D	Diazepam 5mg tab	33.3%	3/9	33%	0/1	0%	½	50%
D	Diazepam 5mg/mL injection	91.7%	8/9	89%	1/1	100%	2/2	100%
D	Diclofenac 50mg tab	83.3%	7/9	78%	1/1	100%	2/2	100%
H	Ergometrine maleate 200mg/mL injection	0.0%	0/9	0%	0/1	0%	0/2	0%
H	Erythromycin 250mg tab	41.7%	2/9	22%	1/1	100%	2/2	100%
C	Ethosuximide 250mg cap/tab	0.0%	0/9	0%	0/1	0%	0/2	0%
D	Fluconazole 200mg cap/tab	50.0%	4/9	44%	0/1	0%	2/2	100%
H	Gentamicin 80mg/2mL injection	100.0%	9/9	100%	1/1	100%	2/2	100%
D	Griseofulvin 125mg tab	41.7%	4/9	44%	0/1	0%	½	50%
D	Hydrochlorothiazide 25mg tab	100.0%	9/9	100%	1/1	100%	2/2	100%
D	Ibuprofen 200mg cap/tab	100.0%	9/9	100%	1/1	100%	2/2	100%
D	Insulin 100 IU/mL soluble injection, 10 mL	25.0%	0/9	0%	1/1	100%	2/2	100%
D	Insulin zinc 100 IU/mL suspension injection, 10 mL	25.0%	0/9	0%	1/1	100%	2/2	100%
H	Magnesium sulphate 50% injection	75.0%	6/9	67%	1/1	100%	2/2	100%
H	Mebendazole 500mg tab	0.0%	0/9	0%	0/1	0%	0/2	0%
D	Metformin 500mg tab	41.7%	2/9	22%	1/1	100%	2/2	100%
D	Methyldopa 250mg tab	75.0%	6/9	67%	1/1	100%	2/2	100%
H	Metronidazole 200mg tab	100.0%	9/9	100%	1/1	100%	2/2	100%
D	Misoprostol 200mcg tab	16.7%	1/9	11%	0/1	0%	½	50%
D	Omeprazole 20mg cap	58.3%	4/9	44%	1/1	100%	2/2	100%
H	Oxytocin 10 IU/mL injection	83.3%	7/9	78%	1/1	100%	2/2	100%
H	Paracetamol 500mg tab	100.0%	9/9	100%	1/1	100%	2/2	100%
H	Paracetamol 24mg/mL suspension	25.0%	2/9	22%	1/1	100%	0/2	0%
H	Paraldehyde injection 10 mL vial	0.0%	0/9	0%	0/1	0%	0/2	0%
H	Phenobarbital sodium 200mg/mL injection	25.0%	1/9	11%	0/1	0%	2/2	100%
H	Phenobarbital sodium 30mg tab	83.3%	7/9	78%	1/1	100%	2/2	100%
D	Phenytoin sodium 50mg/mL injection	0.0%	0/9	0%	0/1	0%	0/2	0%
D	Phenytoin sodium 100mg tab	50.0%	3/9	33%	1/1	100%	2/2	100%
H	Praziquantel 600mg tab	66.7%	6/9	67%	1/1	100%	½	50%
D	Salbutamol inhaler	0.0%	0/9	0%	0/1	0%	0/2	0%
C	Simvastatin 20mg cap/tab	8.3%	0/9	0%	0/1	0%	½	50%
H	Sodium chloride 0.9% injection	67.0%	5/9	56%	1/1	100%	2/2	100%
C	Sodium valproate 200mg cap/tab	25.0%	1/9	11%	0/1	0%	2/2	100%
H	Tetracycline 1% eye ointment	33.3%	4/9	44%	0/1	0%	0/2	0%
H	Zinc sulphate 20mg tab	66.7%	5/9	56%	1/1	100%	2/2	100%

**Fig 2:**
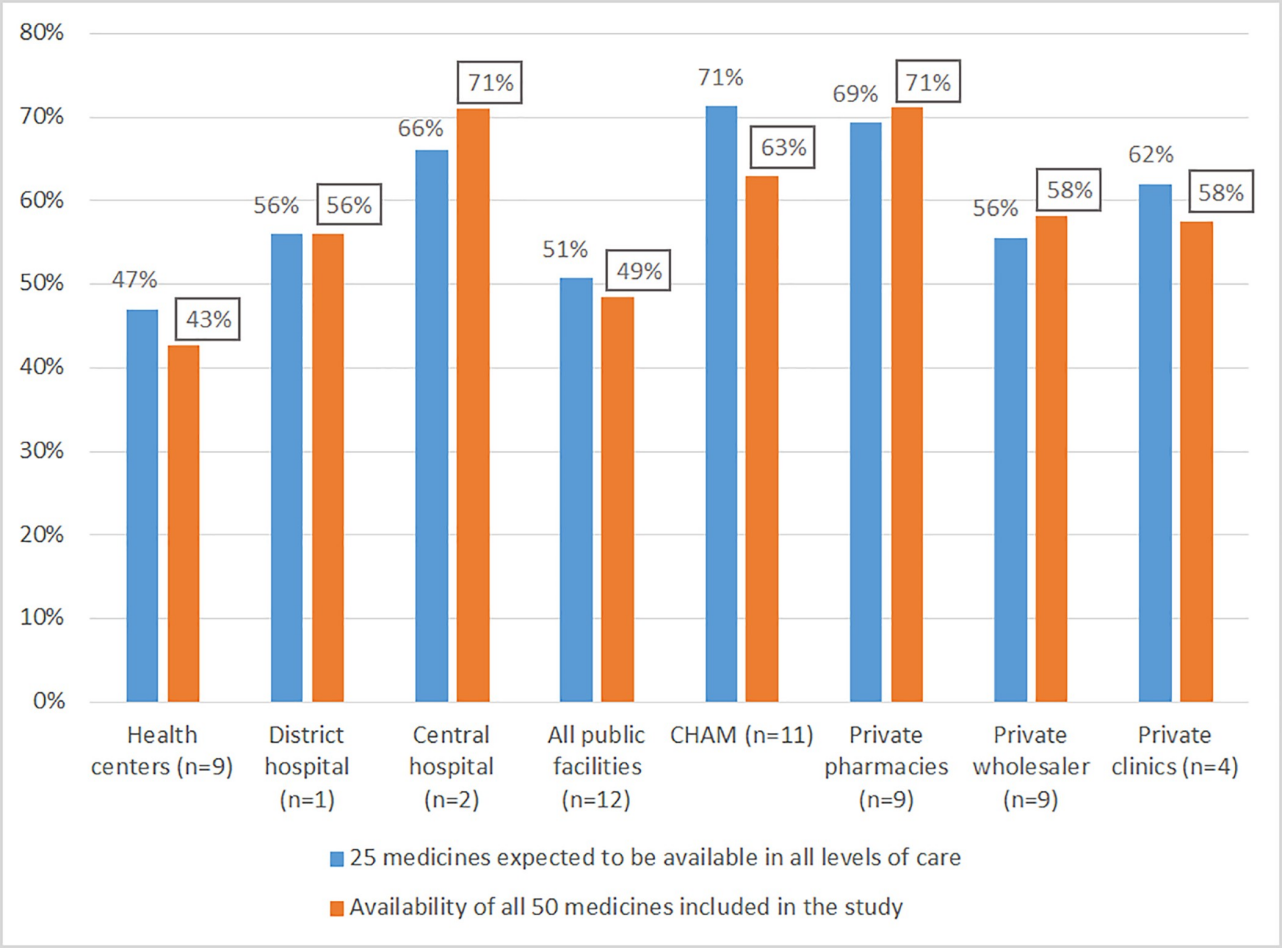
Availability of medicines in Malawi.

Of the 50 medicines included in this study, 25 were supposed to be found in all public health facilities, 19 at district level, five at central level and one (bisoprolol tab/cap) did not have a categorization in the MEML [12] even though it is on WHO/HAI Global core list of medicines. Out of the 50 medicines, nine medicines were available in all public health facilities regardless of levels while eight were unavailable in all public facilities, of which five were for health centre level (Table 2). The higher availability of medicines in the private pharmacies was even more evident with 17 out of 50 medicines being 100% available in this sector (Table 1). There were no substantial differences in the availability of 25 medicines for health centre level and the whole 50 medicines across all sectors (Fig 2).

#### Availability of selected product groups

Availability of essential antibiotics was 100% for amoxicillin and cotrimoxazole tablets/capsules (= caps/tabs) in all facililities. Generally, the availability of the 10 antibiotics included was > 60% in all sectors except for erythromycin tabs (41.7%), amoxicillin suspension (33.3%), cotrimoxazole suspension (16.7%) and tetracycline eye ointment (33.3%) in public facilities–these four products were supposed to be availlable on health centre level (H). On the other hand, two antibiotics (ceftriaxone inj and ciprofloxacin tabs) that were supposed to be found on district (D) and central (C) level only, had a good availablity at health centre level as well (100% and 78% respectively). Private for profit clinics showed 100% availability for all antibiotics except for ceftriaxone (75%), benzylpenicillin (25%) and tetracycline eye ointment (75%).

As already shown for pediatric antibiotic suspensions, the overall availability of pediatric formulations was low in the public sector; paracetamol suspension had an availability of 25%. Availability in CHAM facilities was better for pediatric suspensions (> 60%) and in the private retail pharmacies and clinics a very good availability of up to 100%.

There was a very low availability in the public and CHAM facilities of the anthelmintics albendazole and mebendazole (both 0% in public and 9.1% in CHAM facilities) even though they were both categorized as essential medicines for health centre level. Availability especially for albendazole was better in retail pharmacies at 88.9%.

The availability of maternal medicines (ergometrine inj, magnesium sulphate inj, methyldopa, misoprostol, oxytocin inj) varied from as low as 0% of ergometrine in public facilities and 0% of magnesium sulphate and oxytocin in retail pharmacies to 90.9% availability of oxytocin in public sector facilities (Table 1). Ergometrine was only available in CHAM and private pharmacies. No public sector facility had ergometrine even though it is listed as an essential medicine on MEML. Magnesium sulphate inj and methyldopa were available in 67% and oxytocin in 78% of the public health centres, whereas misoprostol was in only 11%. Notably, of these products only magnesium sulphate and oxytocin are categorized for health centre level.

Of the 9 antiepileptic medicines, three were intended for health centre (H) level (phenobarbital sodium tabs and inj, paraldehyde inj), four were for district level (carbamazepine, diazepam inj, phenytoin sodium tabs and inj) and two for central level (ethosuximide and sodium valproate) according to MEML. Apart from paraldehyde inj, these antiepileptics are listed on the WHO EML as well, however ethosuximide only appears on the complementary list (specialized care necessary). The only “H level” antiepileptic medicine that was widely available was phenobarbital sodium tabs (83% in public facilities, 100% in retail pharmacies and CHAM facilities respectively, but only 50% in private clinics). Phenobarbital inj had a poor (11.1–54%) and paraldehyde a very poor (0% - 9.1%) availability. Availability for these three medicines was generally best in CHAM facilities. Further antiepileptics that were widely available in public and CHAM facilities were carbamazepine (83.3% and 63.3% respectively) and diazepam inj (91.7% and 90.9% respectively) even though these were categorized for district (D) level only. Carbamazepine had 78% and diazepam inj 89% availability in public health centres. Availability for a few antiepileptics was good in retail pharmacies (100% for carbamezepine and phenobarbital sodium), but rather low for other products and also low in private clinics (generally not more than 50%).

A further 11 medicines for NCDs were included in the study. Of those, only one was categorized for health centre level (amitriptyline), the others were for district or central level or had no categorization (bisoprolol). The results were diverse, hydrochlorothiazide being the best available among the antihypertensive medicines (between 88.9% and 100%). Salbutamol inhaler, essential for asthma, was available at none of the public facilities, but in 81.8% of the CHAM facilities and in 100% of the private facilities. Availability of insulin was rather low across all sectors (25% to 55.6%), whereas the oral antidiabetic metformin was better available especially in the private sector (up to 100%).

### Medicine prices in Malawi

The overall median price ratio (MPR) relating to the international reference price was 1.11 in wholesalers (private plus CMST), 2.54 in CHAM facilities, 2.70 in retail pharmacies, and 4.01 in private clinics. Table 3 shows the individual MPR per sector. This showed that the private clinics charged more to the patient than the other sectors, while CHAM facilities were relatively cheaper.

**Table 3:** Median prices in different types of facilities. Prices in US Cents per unit (cap/tab, g, mL, vial).

Level of use according to MEML 2015	Medicine name (including strength and dosage form)	MSH price	Retail pharmacies		CHAM facilities		Private clinics		MPR for wholesalers (private plus CMST)
			Median price	MPR for retail pharmacies	Median price	MPR for CHAM facilities	Median price	MPR for private clinics	
H	Acyclovir 400mg cap/tab	8.35	12.65	1.51	10.33	1.24	25.44	3.05	1.13
H	Acyclovir 5% cream	34.72	8.27	0.24	9.64	0.28	12.50	0.36	0.08
H	Albendazole 10% suspension	0.90	4.13	4.59	5.17	5.74	7.68	8.54	4.59
H	Amitriptyline 25mg tablet	0.84	2.07	2.46	1.86	2.21	3.44	4.10	0.82
H	Amoxicillin 250mg tab/cap	1.60	3.44	2.15	4.13	2.58	4.13	2.58	1.19
H	Amoxicillin 25mg/mL suspension	0.46	1.10	2.40	0.83	1.80	0.96	2.09	1.07
H	Benzylpenicillin 5 MU injection	24.04	86.12	3.58	74.06	3.08	112.30	4.67	1.12
-	Bisoprolol 5mg tab	9.16	13.53	1.48			10.28	1.12	0.81
C	Captopril 25mg tab	2.46	4.13	1.68	2.41	0.98	7.87	3.20	0.55
D	Carbamazepine 200mg tab	1.85	5.10	2.76	4.13	2.23	5.81	3.14	1.03
D	Ceftriaxone 1g powder for injection	39.80	122.63	3.08	117.11	2.94	114.02	2.86	0.95
C	Cimetidine 400mg tab	2.27	5.51	2.43	4.82	2.12	3.54	1.56	1.40
D	Ciprofloxacin 500mg tab	3.73	10.33	2.77	6.89	1.85	8.27	2.22	0.91
H	Cotrimoxazole 480mg tab	1.20	2.48	2.07	2.76	2.30	3.10	2.58	1.03
H	Cotrimoxazole 48mg/mL suspension	0.48	1.10	2.30	0.86	1.79	0.82	1.71	0.85
D	Diazepam 5mg tab	0.96	1.23	4.31	1.72	1.79	2.76	2.87	0.60
D	Diazepam 5mg/mL injection	5.81	34.45	5.93	35.82	6.17	69.79	12.01	1.63
D	Diclofenac 50mg tab	0.45	2.24	4.98	2.76	6.12	3.72	8.27	1.26
H	Erythromycin 250mg tab	3.06	6.89	2.25	6.20	2.03	7.58	2.48	1.29
D	Fluconazole 200mg tab/cap	7.03	21.36	3.04	27.56	3.92	33.99	4.83	2.61
H	Gentamicin 80mg/2mL injection	6.00	13.78	2.30	20.67	3.44	37.32	6.22	0.86
D	Griseofulvin 125mg tab	1.78	4.82	2.71	4.82	2.71	11.44	6.42	1.34
D	Hydrochlorothiazide 25mg tab	0.43	0.92	2.14	1.38	3.20	3.10	7.21	0.68
D	Ibuprofen 200mg tab/cap	0.68	1.23	1.80	2.07	3.04	2.24	3.29	0.77
D	Insulin 100 IU/mL soluble injection, 10 mL	59.22	206.68	3.49	107.47	1.81	130.90	2.21	1.66
D	Insulin zinc 100 IU/mL suspension injection, 10 mL	111.40	245.26	2.20	168.79	1.52	130.90	1.18	0.45
H	Magnesium sulphate 50% injection	7.34			37.20	5.07	94.38	12.86	0.92
D	Metformin 500mg tab	1.50	3.44	2.30	2.76	1.84	5.51	3.67	0.82
D	Methyldopa 250mg tab	3.24	7.23	2.23	5.51	1.70	5.74	1.77	1.10
H	Metronidazole 200mg tab	0.61	1.84	3.01	2.76	4.52	2.66	4.37	1.02
D	Misoprostol 200mcg tab	18.10	41.34	2.28	22.39	1.24	89.56	4.95	2.28
D	Omeprazole 20mg cap	1.41	4.48	3.18	4.13	2.93	5.86	4.15	0.80
H	Oxytocin 10 IU/mL injection	15.57			55.11	3.54	41.34	2.65	1.28
H	Paracetamol 500mg tab	0.44	1.15	2.61	2.07	4.70	2.07	4.70	1.18
H	Paracetamol 24mg/mL suspension	0.54	0.83	1.53	0.83	1.53	0.81	1.51	0.74
H	Phenobarbital sodium 200mg/mL injection	381.33	165.34	0.43	71.65	0.19	133.38	0.35	0.21
H	Phenobarbital 30mg tab	0.75	1.38	1.84	1.38	1.84	2.27	3.03	0.81
D	Phenytoin 100mg tab	1.04	8.61	8.28	0.69	0.66			0.72
H	Praziquantel 600mg tab	10.99	33.07	3.01	20.67	1.88	55.11	5.01	1.06
D	Salbutamol inhaler	0.92	2.07	2.25	1.38	1.50	2.07	2.25	0.97
C	Simvastatin 20mg tab/cap	5.25	13.53	2.58					1.12
H	Sodium chloride 0.9% injection	0.10	0.28	2.76	0.24	2.41	0.34	3.45	1.17
C	Sodium valproate 200mg tab/cap	6.95	13.09	1.88	6.89	0.99	52.17	7.51	0.90
H	Tetracycline 1% eye ointment	5.12	12.80	2.50	13.78	2.69	13.78	2.69	1.15
H	Zinc sulphate 20mg tab	1.41	4.13	2.93	4.13	2.93	9.64	6.84	0.94

Of the paediatric formulations in the study, paracetamol syrup had the lowest MPR across the sectors with MPRs of 0.74 for wholesale, 1.51 for private clinics and 1.53 for both CHAM and private pharmacies. Amoxicillin and cotrimoxazole suspensions had lower MPR in CHAM facilities than in private pharmacies. However, cotrimoxazole suspension had the lowest pricing in the private clinics compared to the other sectors, with MPR of 1.71 against 1.79 and 2.30 for CHAM and retail pharmacies respectively.

Except for metronidazole, the oral antibiotic formulations for adult patients (amoxicillin, cotrimoxazole, ciprofloxacin, and erythromycin) showed relatively low MPRs and two of those (amoxicillin, cotrimoxazole) had even lower MPRs at retail pharmacies than in CHAM and private clinics (Table 3). Metronidazole tablets had a higher MPR across all sectors, between 3.01 at retail pharmacies and 4.52 at CHAM facilities.

The MPR for antiepileptic medicines varied quite a lot between the medicines and sectors. For diazepam inj, MPR at wholesalers was 1.63 and increased to 5.93 in retail pharmacies, 6.17 in CHAM and 12.01 in private clinics. Apart from phenytoin sodium tabs in retail pharmacies (MPR 8.28) and sodium valproate tabs in private clinics (MPR 7.51), all other MPRs for antiepileptics were between 0.19 and 3.14. MPRs for ethosuximide inj, phenytoin inj and paraladehyde inj could not be calculated, as these products were available in too few of the facilities (<2).

### Affordability of medicines in Malawi

**Fig 3:**
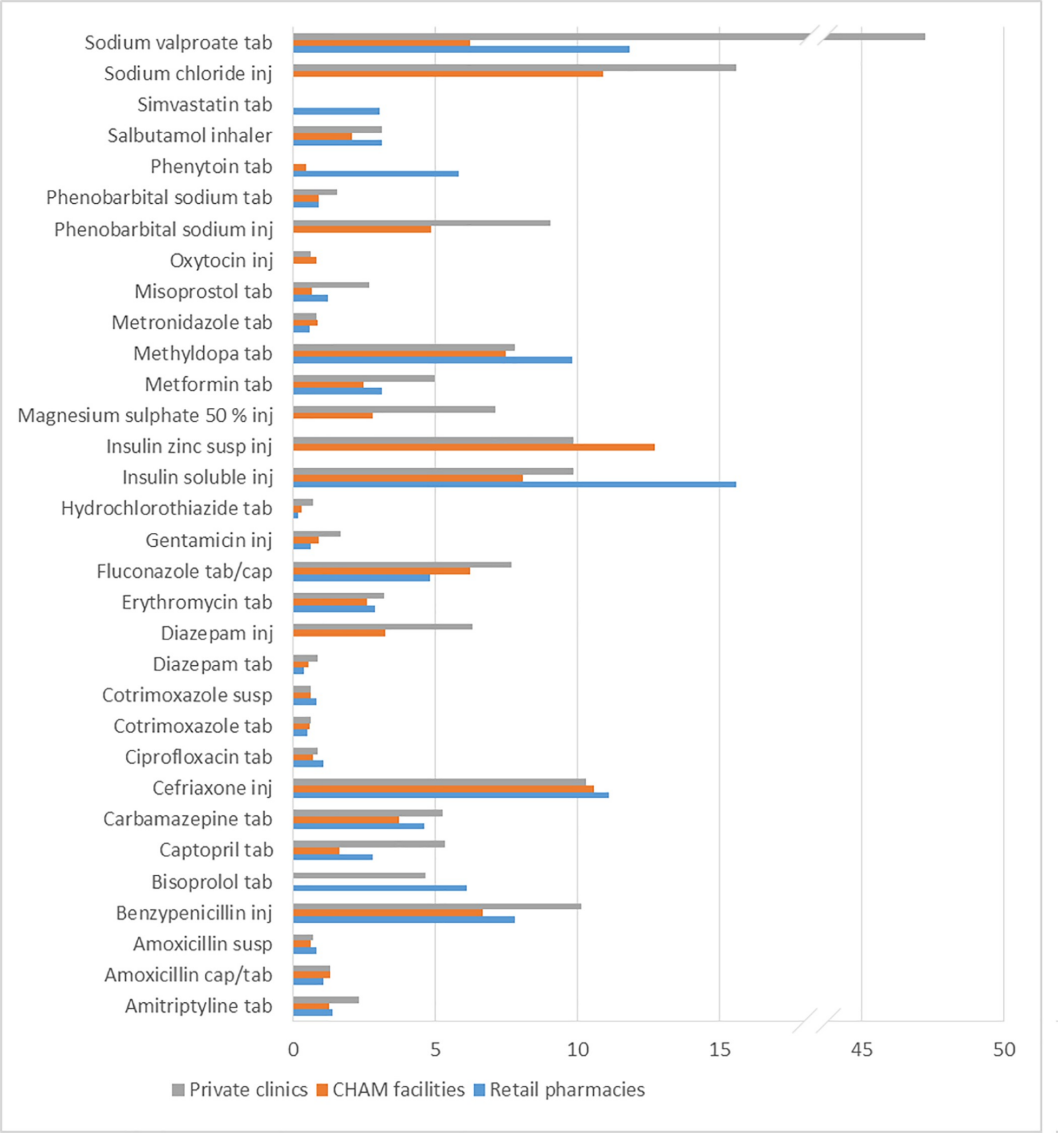
Affordability of medicines priority diseases in Malawi. The number of day's wages needed to purchase a course of treatment was calculated from the daily salary of the lowest-paid unskilled government worker (1.33 US $). See Methods for calculation.

The affordability of medicines was estimated using WHO/HAI methodology and course of treatment using MSTG 2015. The affordability has been calculated based on the priority diseases of Malawi derived from the WHO’s burden of disease and the Ministry of Health Malawi (see methods section for the diseases selected). The results are shown in Fig 3 below. Since there were differences in median prices between retail pharmacies, CHAM and private clinics/hospitals the results have been split. Of the 32 medicines for priority diseases, 18 medicines exceeded the Malawian daily wage for the cost of one course of treatment across all sectors, thus making them unaffordable to many people.

Of special note is that the commonly used oral antibiotics for adults and children were mostly affordable. For instance, cotrimoxazole and amoxicillin suspensions which are frequently prescribed for children under five needed maximum 0.8 days to work to afford the treatment even in the retail pharmacies. Gentamicin injection, metronidazole and ciprofloxacin which are also recommended for sexually transmitted diseases, were all affordable across the sectors with only gentamicin being slightly more expensive in private clinics needing 1.7 days of work to afford.

Concerning NCDs, it could be seen that methyldopa (used for hypertension in pregnant women) and insulin preparations were little affordable in CHAM facilities and the private sector, with more than 7 days`work necessary to afford the full course of monthly treatment. Insulin soluble for type 1 diabetes, was the most expensive of all the NCD medicines across all sectors requiring 15.6 days, 8.1 days and 9.9 days of work to afford in retail pharmacies, CHAM and private clinics respectively. Metformin which is used as first-line treatment for type 2 diabetes required a lower number of working days for CHAM (2.5 days) and more days in private clinics (5.0 days). Hydrochlorothiazide was the most affordable NCD medicine requiring less than a day’s wage to purchase a maintenance dose for one month. Apart from hydrochlorothiazide which is the main medicine used for hypertension in Malawi, other antihypertensive medicines were less affordable (captopril, methyldopa and bisoprolol). For a salbutamol inhaler, an asthmatic patient required 2.1 days of work for a monthly treatment from CHAM facilities, or 3.1 days at private clinics and retail pharmacies.

Affordability of antiepileptics was in general very poor with sodium valproate being most expensive across all sectors. Sodium valproate needed 47.2 days of work for a patient to purchase the medication in private clinics, 11.8 days in retail pharmacies and 6.2 days from CHAM facilities. Only the treatment with phenobarbital sodium tabs required less than one day of work for a monthly maintenance course and thus is considered affordable. (See Fig 3 for details.)

Of maternal products (oxytocin, misoprostol, magnesium sulphate injection, and methyldopa), oxytocin was highly affordable as it required less than a day’s wage to purchase a treatment course in both CHAM and private clinics. Misoprostol, mainly used for PPH was cheapest in the CHAM facilities requiring less than a day’s wage for a full course of treatment or 1.2 and 2.7 days in retail pharmacies and private clinics respectively. Magnesium sulphate injection for pre-eclampsia and eclampsia in pregnancy required 2.8 days of work in CHAM and 7.1 days of work to afford in the private clinics, without additions of costs associated with the administration of the medicine, and is thus unaffordable (Fig 3).

The calculation of treatment courses and affordability data for all medicines included in the data analysis is displayed in S4 Table.

## Discussion

### Availability of medicines in Malawi

Our study has shown that the availability of essential medicines in Malawi is generally higher in the private for-profit and non-profit sectors than in the public sector. This goes hand in hand with the findings of studies in a range of other developing countries where availability in the private sector was usually highest [23]. The higher availability of medicines in the private sector may have to do with efficiency and profit maximization of the sector as seen in a market economy. The poor availability of medicines in the public sector may reflect inadequate government funding to the health sector. This is evidenced by the continuously increasing reliance on donor funding for the health sector. In 2012, the most recent year for which data are available, donor funding contributed 68% of total health expenditure, while funding by the government of Malawi contributed only 16.1% [24]. However, the overall availability in all sectors falls short of the recommended minimum availability of 80% as set by the WHO [15, 16]. Furthermore, the picture was rather diverse concerning different product groups and individual products in our study.

Malawi as a country is doing well in the availability of adult antibiotics, especially amoxicillin, ciprofloxacin, cotrimoxazole tablets, gentamicin injections and metronidazole tablets which showed an availability of more than 80% in all sectors as recommended by the WHO [15, 16]. This is better than the findings of surveys executed in Kenya, Namibia, Rwanda, Tanzania and Uganda, including both public and faith-based facilities, which showed on average 70% availability of amoxicillin caps/tabs, 28% of ceftriaxone, and 62% of benzyl penicillin injections [25]. Our recent study [11] shows an availability of amoxicillin tablets of only 40% in CHAM and 75% in public facilities, thus the increase to 100% availability in all sectors for this essential antibiotic is important as it will help to fight communicable diseases in the country.

However, the poor availability of amoxicillin (33.3%) and cotrimoxazole (16.67%) suspensions in public facilities is a matter of concern. This is contrary to other low income countries like Ethiopia, where cotrimoxazole suspension was available in 100% of public facilities [26]. The low availability of cotrimoxazole suspension may be due to insufficient categorization in the MEML 2015 [14], where only ‘level of use’ for the product was indicated but no ‘therapeutic priority’ and ‘procurement system’, making it appear not to be a priority in Malawi. This creates problems in the dosage for children under the age of five as they are forced to use adult tablets. Despite the poor availability of paediatric antibiotics in public facilities, there was good availability of these medicines in retail pharmacies, CHAM and private clinics. The pediatric antibiotic syrups were not only available, but also affordable across the whole private sector as it required less than a day’s wage to purchase the full treatment.

Ciprofloxacin and ceftriaxone injections are mainly supposed to be available in secondary facilities, however there was a high availability of 83.3% and 100% respectively in all public facilities including the primary facilities. The availability of ciprofloxacin may be explained by its widespread use with sexually transmitted diseases. However, the high availability of ciprofloxacin in primary health facilities may lead to unnecessary use resulting in the promotion of antimicrobial resistance [27]. Ceftriaxone injection is a third generation cephalosporin which is mainly used to treat conditions with resistance to other antibiotics. Its 100% availability in the public primary health facilities is a cause for worry. At the same time, it was widely available in the private and CHAM sector making it prone to indiscriminate use in Malawi. This can promote the emergence of resistant pathogens in the country [28–30]. In the face of growing concerns about antimicrobial resistance, policy makers need to regulate the availability of these antibiotics in lower level health facilities. Knowing that for the past 20 to 30 years, there have been very few new antibiotic drug discoveries [31], there is a need to preserve the few antibiotics we have.

Looking at maternal health products in our study, according to MEML, magnesium sulphate inj, oxytocin inj and ergometrine inj were to be found on health centre level, whereas methyldopa and misoprostol were indicated for district level only. Whereas availability of magnesium sulphate inj and oxytocin was not very low for CHAM and public facilities (between 72.7% and 90.9%), it was only 50% in private clinics. However, for these life-saving and quickly needed products, an availability of 100% should be the target for all facilities where births are taking place. Even though ergometrine injection is still included in the MEML for treatment of PPH, it was unavailable in any of the public health facilities. It was found, however, in some of the CHAM facilities and retail pharmacies. There is a need for a wider review of the use of ergometrine on PPH. As the international (WHO) guidelines of treatment for PPH have changed and generally do not recommend the use of ergometrine anymore[32], there is a need for the government of Malawi to update the health workers and MSTG/MEML accordingly.

Concerning NCDs, the big range of antiepileptics included in the study gave a comprehensive picture on treatment availability for this often neglected disease. High availability of phenobarbital tabs in public and CHAM facilities is a good sign, as this has been reported to be the most widely used [33] and most cost-effective antiepileptic medicine in the developing world [34]. Carbamazepine, categorized as district-level medicine only, was available in 78% of the public health centres (and in 63.6% of CHAM facilities) and can serve as an important treatment option, even though its availability falls short of the recommendations by WHO [15, 16]. The same is valid for diazepam inj used for emergency cases which had a high availability (>90%) in public and CHAM facilities but a very poor availability in the private sector. The poor availability of diazepam inj in retail pharmacies may be due to the nature of the medicine because it is mostly administered in emergency situations as well as a sedative in surgery where retail pharmacies are not involved. On the other hand, the poor availability of H-level medicines phenobarbital inj and paraldehyde inj on health centre level may not be as critical if diazepam inj is available for emergencies (status epilepticus). Overall availability of antiepileptic medicines was better than reported from 46 countries in 2012 by Cameron et al [35] where they found availability of <50% for all antiepileptics except diazepam. If antiepileptic treatment can be administered by skilled health workers on health centre level, it is positive to roll out treatment to this level. However, categorization in the MEML should correspond to the actual policy.

Only a smaller range of further NCD medicines was included in the study, therefore general statements on availability of treatment for NCDs other than epilepsy are limited. Concerning antihypertensives, only one product (hydrochlorothiazide) was widely available. Two further antihypertensives (bisoprolol and captopril) were included because they belong to the WHO/HAI Global core list of medicines, but they do not represent the first treatment options of the respective groups in Malawi. Instead, atenolol and enalapril are recommended by MEML, this is also based on WHO EML recommendations. It is interesting to see that all antihypertensive medicines listed on the MEML are categorized only for district- or central-level use, not for health centre level. This may be due to the fact that opportunities for diagnosis and monitoring are limited on the health centre level because skilled personnel is scarce. A similar situation was seen for the treatment of asthma, where salbutamol inhalers were unavailable in all public facilities but widely available in CHAM facilities (81.8%) and the private sector (100%). The recommended medicines for health centre level, in this case aminophylline tabs, salbutamol tabs and adrenaline inj, however, were not included in the study. In order to face the increasing challenge of NCDs, it would be desirable to roll out NCD treatment and train health workers accordingly.

### Medicine prices in Malawi and policy implications

The study in Malawi showed that the average MPR was 2.70, 2.54 and 4.01 for the retail pharmacies, CHAM and private clinics/hospital respectively. It was interesting to note that prices in the private clinics were higher than those of retail pharmacies and CHAM, but not anywhere close to the higher MPR of 5.37 found in secondary analysis of 36 developing and middle-income countries [36]. However, medicine prices in CHAM facilities may be lower due to some donations they receive from various international organizations as well as to having access to the Central Medical Stores Trust to get their supplies.

In terms of wholesale pricing, the overall MPR of 1.11 is good and lower than expected from the majority of low and middle income countries, such as Malaysia in which an MPR of 2.41 was found in 2007 [37]. Of special note is that out of the whole 50 medicines included in this study, only three medicines had an MPR higher than 2.0 at wholesale level. These medicines are albendazole, misoprostol and fluconazole with MPRs of 4.59, 2.28 and 2.61 respectively as shown in Table 3.

The private clinics showed generally higher MPRs which is due to the profit maximization characteristic of this sector. However, even though the private sector had higher MPR, in comparison to other countries [35–38], the prices were considerably lower. The favourable prices observed in the private sector in Malawi are consistent with the experience of the authors of this study. They may result, at least in part, from the fact that medicines are provided free of charge in the public health care system, creating a competitive environment. In the private sector of many countries, prices are often inflated by the high cost of originator brand medicines which was not the case in this study. For continued favourable prices in Malawi, the continued provision of free services in the public sector will be ideal.

### Affordability of medicines in Malawi and policy implications

The cost of medicine is considered affordable if it amounts to the wage of one workday (of the lowest paid government worker) or less, for one course of treatment [13]. By this criterion, 18 out of 32 treatments shown in Fig 3 are still unaffordable for a large part of Malawi’s population. This coincides with the results of a recent study in Malawi, reporting that medical costs at CHAM and private facilities are considered as the main barrier to health care by the population. [39]. Our affordability assessment is based on the currently common treatment options as advocated by the Ministry of Health in the public sector.

Of the medications listed in Fig 3, cotrimoxazole tabs, cotrimoxazole syrup, amoxicillin syrup, ciprofloxacin tabs, metronidazole tabs and gentamicin inj are the most affordable antibiotics, across all sectors. It is certainly not a coincidence that these four most affordable antibiotics are also the ones which are most available in all sectors (except for the very poor availability of the syrups in the public sector). As mentioned above, ciprofloxacin was much more widely available than warranted by the provisions of the Malawi Essential Medicines List [40, 41].

Of all antibiotics listed in Fig 3, ceftriaxone and benzylpenicillin showed the lowest affordability across all sectors. This may be attributed to the need for administration charges as well as other accessories (syringes, water for injection etc.). However, the cost thereof was high in the retail pharmacies, too, even though they do not administer the product directly to the patient. This should be a cause for worry because if CHAM facilities and private clinics do not have the product, the cost of it may escalate as the patients may have to purchase from the pharmacies and then be charged extra for administration at a nearby clinic. A recent study in Tanzania proved that the cost of treatments was the most important of all factors which determine whether a patient receives appropriate or inappropriate treatment [42]. Treatment could be optimized by an improvement of the affordability of medications.

Concerning NCDs, the least affordable is insulin therapy for diabetes patients which can consume up to 15.6 days’ worth of wages at a retail pharmacy. But also oral metformin was still at a cost of two days’ wages, thus considered unaffordable, as well. Similarly, simvastatin, salbutamol inhaler, methyldopa, captopril and bisoprolol were unaffordable to the population (Fig 3). Methyldopa was consistently expensive across all sectors requiring a minimum of seven days’ worth of work for a lowest paid worker.

Among the antiepileptic medicines, phenobarbital tab was the only affordable medicine with less than one day’s work necessary. Luckily, this was also the best available antiepileptic medicine and represents the first treatment option in developing countries. All other antiepileptic medicines, however, had a poor affordability needing between three and twelve days’ work. As epilepsy is still a stigmatized disease and is linked with poverty if patients cannot earn a regular income [43], it is very important to have these products available free of charge in the public sector.

Due to unaffordability of more medicines in Malawi, there is a need to delay the introduction of cost recovery for medicines as envisioned by the Malawi National Medicine Policy [7]. The unaffordability of medicines in Malawi is partly attributed to medicine prices, but the most important contributing factor is the low income of the lowest paid workers [22], who in most cases cannot afford the treatment. Thus, in the short term, a delay in the introduction of medication charges will cushion the poor in Malawi, while at the same time, the government of Malawi, together with various labour organizations in the country, should strive to increase minimum wages.

The unaffordability of medicines coupled with poor availability of medicines in the public sector needs serious attention by the government of Malawi. It is good to note that in the Malawi Growth and Development Strategy III, the issues of availability and financing of health care have been included. Among these strategies, the Malawi government has now included plans to increase finances to the health sector through increasing direct budgetary allocation, as well as exploring user fees and insurance schemes as some of the measures to ensure sustainability of health care provision [44]. As one of the measures to minimize concerns about affordability, the Malawi government plans to expand access to essential medicines in the CHAM sector by expanding service level agreements (SLAs) to more CHAM facilities, with a further option of trying similar agreements with the private sector [44]. SLAs ensure that maternal and under-five child health services offered in selected CHAM facilities are provided free of charge to the surrounding communities [44, 45]. The provision of free health care to pregnant women and children helps in bolstering the affordability of health services in CHAM facilities. However, implementation of these plans would require increased financial resources from taxation as well as from international partners, which requires careful planning by the government.

### Limitations

One limitation of the study was the poor response from the private clinics which reduced the amount of data collected. However, considering that this sector serves only approximately 3% of the population, the lack of data from this sector does not have much impact on our assessment of the availability and affordability of medicines in Malawi.

Secondly, the focus of the study was on two cities. Due to limited resources, it was not possible to include further districts and more sampling sites.

The third limitation was the unavailability of originator brands in the sampled facilities which forced us to deviate from WHO/HAI methodology. However, this in itself did not have an impact on either the results of availability in general or the affordability.

## Conclusion

Our study has shown low availability of essential medicines in the public sector especially for pediatric dosage formulations and for a range of NCD medications. Overall, the picture was rather diverse depending on product and sector. Availability of adult formulation antibiotics was quite high, thus supporting the fight against communicable diseases which are ravaging Malawi. The higher availability of antibiotics should however be checked by MoH and other policy makers in view of the increasing cases of antimicrobial resistance.

Medicine pricing in Malawi is favourable as compared to other low income countries, however affordability of medicines in the private sector and faith-based facilities is a challenge. With the low earning power of the majority of Malawians, we recommend the continued provision of free medical care in the public sector and subsidization of treatment in the faith-based sector (CHAM) to avoid catastrophic expenditure for the majority of Malawians who are living below the poverty line.

## Supporting information

S1 Table List of tracer medicines for measuring stockouts in Malawi. (PDF)

S2 Table Justification for the choice of medicines. (PDF)

S3 Table Medicine price data collection form. (PDF)

S4 Table Calculation of medicine treatment costs and affordability. (PDF)
