# Supplementary material for: The availability, prices and affordability of essential medicines in Malawi: A cross-sectional study
Source: PLoS One. 2019 Feb 12;14(2):e0212125. doi: 10.1371/journal.pone.0212125 (PMC6372227; doi:10.1371/journal.pone.0212125)
Supplement: S1 Table — (PDF) [file pone.0212125.s001.pdf]

S1 Table. List of Tracer Medicines for Measuring Stockout by Ministry of Health Malawi.

Name of medicine	Whether included in this study or not	Reasons for exclusion
Lumefantrine/Artemether 20/120 mg - 6x1	Not selected	Provided through donation
Lumefantrine/Artemether 20/120 mg - 6x4	Not selected	Provided through donation
Malaria Rapid Diagnostic Test Kits	Not selected	Laboratory or surgical items and not the focus of the study
Artesunate Injection 60mg	Not selected	Provided through donation
Magnesium sulphate 50%, 2ml ampoule	Selected	
Male Condoms	Not selected	Provided through donation
Medroxyprogesterone acetate inj, 150mg/ml (DEPOPROVERA)	Not selected	Provided through donation
Oxytocin 10 IU/ml, 1ml	Selected	
Amoxycillin 125mg/5ml suspension	Selected	
Oral rehydration salt, satchet (WHO formula) for 1L solution	Not selected	
Tetracycline eye ointment 1%, 3.5g/5gm	Selected	
Gentamicin 40mg/ml, 2ml	Selected	
Benzylpenicillin 3g (5MU), PFR	Selected	
Determine HIV Test Kits	Not selected	Provided through donation
Tenofovir (TDF) + Lamivudine (3TC) + Efavirenz (EFV), 300+300+600mg, 30's (5A)	Not selected	Provided through donation
RH 150/75	Not selected	Provided through donation
RH 60/60	Not selected	Provided through donation
Streptomycin 1g	Not selected	Provided through donation
Cotrimoxazole 480mg	Selected	
Dextrose (glucose) 5%, 500ml	Not selected	
Diazepam 5mg/ml, 2ml	Selected	
Glove disposable powdered latex large, 100 pieces	Not selected	Laboratory or surgical items and not the focus of the study
Glove disposable powdered latex medium, 100 pieces	Not selected	Laboratory or surgical items and not the focus of the study
Glove surgeon's size 7½ sterile, pair	Not selected	Laboratory or surgical items and not the focus of the study
Metronidazole 200mg	Selected	
Sodium Chloride injectable 0.9% 500ml	Selected	
Syringe, autodestruct, 2ml, disposable, hypoluer with 23g needle	Not selected	Laboratory or surgical items and not the focus of the study
Syringe, autodestruct, 5ml, disposable, hypoluer with 21g needle	Not selected	Laboratory or surgical items and not the focus of the study
Amoxycillin 250mg	Selected	
