# Supplementary material for: The availability, prices and affordability of essential medicines in Malawi: A cross-sectional study
Source: PLoS One. 2019 Feb 12;14(2):e0212125. doi: 10.1371/journal.pone.0212125 (PMC6372227; doi:10.1371/journal.pone.0212125)
Supplement: S2 Table — (PDF) [file pone.0212125.s002.pdf]

S2 Table. Justification for choice of Medicines.

	Disease	Medicine	Reason for inclusion
1	Antiherpes	Acyclovir 400mg cap/tab	Essential medicine on Malawi Essential Medicine List (MEML)
2	Antiherpes	Acyclovir 5% cream	Essential medicine on MEML
3	Anthelmintic	Albendazole 10% suspension	Essential medicine on MEML and to be available in all health facilities
4	Depression	Amitriptyline 25mg tab	Essential medicine on WHO-HAI Global list of medicines.<br>Also essential in MEML
5	Infectious disease	Amoxicillin 250mg cap/tab	Medicine is on the List of Tracer Medicines for measuring Stock-outs by Ministry of Health-Malawi. As well as used for post-partum haemorrhage It is also on WHO-HAI global list
6	Infectious disease	Amoxicillin 25mg/mL suspension	Medicine is on the List of Tracer Medicines for measuring Stock-outs by Ministry of Health-Malawi. It is also on WHO-HAI global list
7	Cardiovascular disease	Benzylpenicillin 5 MU injection	Medicine is on the List of Tracer Medicines for measuring Stock-outs by Ministry of Health-Malawi and used in obstetrics
8	Infectious disease	Bisoprolol 5mg tab	Essential medicine on WHO-HAI Global list of medicines as well as essential on MEML
9	Cardiovascular diseases	Captopril 25mg tab	Essential medicine on WHO-HAI Global list of medicines
10	CNS	Carbamazepine 200mg tab	Used as anticonvulsant in Malawi.
11	Infectious disease	Ceftriaxone 1 g in vial, powder for injection	Essential medicine on WHO-HAI Global list of medicines
12	Infectious disease	Cimetidine 400mg tab	Essential medicine in MEML
13	Ulcer	Ciprofloxacin 500mg tab	Essential medicine on WHO-HAI Global list of medicines
14	Infectious disease	Cotrimoxazole 480mg tab	Medicine is on the List of Tracer Medicines for measuring Stock-outs by Ministry of Health-Malawi
15	Infectious disease	Cotrimoxazole 48mg/mL suspension	Essential medicine on WHO-HAI Global list of medicines
16	CNS	Diazepam 5mg tab	Medicine is on the List of Tracer Medicines for measuring Stock-outs by Ministry of Health-Malawi and used in obstetrics
17	CNS	Diazepam 5mg/mL injection	Medicine is on the List of Tracer Medicines for measuring Stock-outs by Ministry of Health-Malawi and used in obstetrics
18	Pain/ inflammation	Diclofenac 50mg tab	Essential medicine on WHO-HAI Global list of medicines
19	Maternal	Ergometrine maleate 200mg/mL injection	Indicated for use as an oxytocics in MEML
20	Infectious disease	Erythromycin 250mg tab	Essential medicine for sexually transmitted disease (STD) to be available in all health facilities.
21	CNS	Ethosuximide 250mg cap/tab	Used as anticonvulsant in Malawi.

	**Disease**	**Medicine**	**Reason for inclusion**
22	Antifungal	Fluconazole 200mg cap/tab	Essential medicine on MEML.
23	Infectious disease	Gentamicin 80mg/2mL injection	Medicine is on the List of Tracer Medicines for measuring Stock-outs by Ministry of Health-Malawi
24	Diabetes	Griseofulvin 125mg tab	Essential medicine on MEML
25	Antifungal	Hydrochlorothiazide 25mg tab	Essential medicine on MEML
26	Cardiovascular	Ibuprofen 200mg cap/tab	Essential medicine on MEML
27	Pain/ inflammation	Insulin 100 IU/mL soluble injection, 10 mL	Essential medicine on MEML and consumption is increasing due to increase in NCD's
28	Diabetes-Insulins	Insulin zinc 100 IU/mL suspension injection, 10 mL	Essential medicine on MEML and consumption is increasing due to increase in NCD's
29	Diabetes-Insulins	Magnesium sulphate 50% injection	Medicine is on the List of Tracer Medicines for measuring Stock-outs by Ministry of Health-Malawi
30	Pre-Eclampsia, Eclampsia	Mebendazole 500mg tab	Essential medicine on MEML to be available in all facilities
31	Anthelmintic	Metformin 500mg tab	Essential medicine on WHO-HAI Global list of medicines. Essential medicine in MEML. First-line treatment for non-insulin dependent diabetes
32	Cardiovascular	Methyldopa 250mg tab	Essential medicine on MEML and highly recommended as anti-hypertensive for pregnant women
33	Infectious disease	Metronidazole 200mg tab	Medicine is on the List of Tracer Medicines for measuring Stock-outs by Ministry of Health-Malawi
34	Oxytocics	Misoprostol 200mcg tab	Indicated for use as an oxytocic in MEML
35	Ulcer	Omeprazole 20mg cap	Essential medicine on WHO-HAI Global list of medicines.<br>Essential medicine for ulcers in MEML
36	Oxytocics	Oxytocin 10 IU/mL injection	Medicine is on the List of Tracer Medicines for measuring Stock-outs by Ministry of Health-Malawi
37	Pain/ inflammation	Paracetamol 500mg tab	Indicated for use in all health facilities in MEML
38	Pain/ inflammation	Paracetamol 24mg/mL suspension	Essential medicine on WHO-HAI Global list of medicines
39	Anticonvulsant	Paraldehyde injection 10 mL vial	Indicated for use in all health facilities as anticonvulsant in MEML
40	Anticonvulsant	Phenobarbital sodium 200mg/mL injection	Indicated for use in all health facilities as anticonvulsant in MEML
41	Anticonvulsant	Phenobarbital sodium 30mg tab	Indicated for use in all health facilities as anticonvulsant in MEML
42	Anticonvulsant	Phenytoin sodium 50mg/mL injection	Indicated for use as anticonvulsant in Malawi Essential Medicines List
43	Anticonvulsant	Phenytoin sodium 100mg tab	Indicated for use as anticonvulsant in Malawi Essential Medicines List
44	Antischistosomal	Praziquantel 600mg tab	Essential medicine on MEML. The only medicine for bilharzia in Malawi
45	Asthma	Salbutamol inhaler	Essential medicine on WHO-HAI Global list of medicines

	Disease	Medicine	Reason for inclusion
46	Cardiovascular disease	Simvastatin 20mg cap/tab	Essential medicine on WHO-HAI Global list of medicines. Also available in MEML as lipid-lowering medicine
47		Sodium chloride 0.9% injection	Medicine is on the List of Tracer Medicines for measuring Stock-outs by Ministry of Health-Malawi and used in obstetrics
48	Anticonvulsant	Sodium valproate 200mg cap/tab	Indicated for use as anticonvulsant in Malawi Essential Medicines List
49		Tetracycline 1% eye ointment	Medicine is on the List of Tracer
50	Diarrhea	Zinc sulphate 20mg tab	Medicine is on the List of Tracer
