# Supplementary material for: The availability, prices and affordability of essential medicines in Malawi: A cross-sectional study
Source: PLoS One. 2019 Feb 12;14(2):e0212125. doi: 10.1371/journal.pone.0212125 (PMC6372227; doi:10.1371/journal.pone.0212125)
Supplement: S3 Table — (PDF) [file pone.0212125.s003.pdf]

College of Medicine-Pharmacy Department

A SURVEY ON AVAILABILITY AND AFFORDABILITY OF ESSENTIAL MEDICINES IN SOUTH AND CENTRAL REGIONS OF MALAWI

Principal Investigator: Felix Khuluza Co-Investigator: Christine Haefele-Abah

Medicine Price Data Collection Form

Use a separate form for each medicine outlet

COMREC #: P.03/17/2131
PMPB#: PMPB/PFK/7

Date : ________________ Survey area number : _______________

Name of town/village/district :

Name of medicine outlet (optional):

Medicine outlet unique survey ID (mandatory):

Distance in km from nearest town (population >50 000):

Type of medicine outlet :

☐ Public sector facility (specify level of care below):
- ☐ Primary care facility
- ☐ Secondary care facility
- ☐ Tertiary care facility

☐ Private sector medicine outlet

☐ Other sector medicine outlet (please specify): __________________

Type of price :
☐ Procurement price ☐ Price the patient pays

Type of data:
☐ Sample outlet ☐ back-up outlet ☐ validation visit

Name of manager of the medicine outlet (Optional-seek consent first):

Name of person(s) who provided information on medicine prices and availability (if different from manager):

Name of data collectors :

Verification

To be completed by the area supervisor at the end of the day, once data have been verified. Signed:___________Date:___________________________

Medicine Price Data Collection Form

Lowest priced generic equivalent product: determined at facility

A	B	C	D	E	F	G	H	I	J
**Generic name, dosage form, strength**	**Medicine Type**	**Brand or product name(s)**	**Manufacturer**	**Available yes/no**	**Pack size recommended**	**Pack size found**	**Price of pack found**	**Unit price (4 decimal places)**	**Comments**
Acyclovir 400 mg cap/tab	Highest priced generic				100			per cap/tab	
	Most sold generic				100			per cap/tab	
	Lowest-priced generic				100			per cap/tab	
Acyclovir cream 5% 10G	Highest priced generic				10 G			per gram	
	Most sold generic				10 G			per gram	
	Lowest-priced generic				10 G			per gram	
Albendazole Susp. 10% 20ML	Highest priced generic				20			per mililitre.	
	Most sold generic				20			per mililitre	
	Lowest-priced generic				20			per mililitre.	

Amitriptyline 25 mg cap/tab	Highest priced generic				100			per cap/tab	
	Most sold generic				100			per cap/tab	
	Lowest-priced generic				100			per cap/tab	
Amoxicillin 250 mg cap/tab	Highest priced generic				1000			per cap/tab	
	Most sold generic				1000			per cap/tab	
	Lowest-priced generic				1000			per cap/tab	
Amoxicillin 25 mg/ ml Susp.	Highest priced generic				100 ML			per mililitre	
	Most sold generic				100 ML			per mililitre	
	Lowest-priced generic				100 ML			per mililitre	
Benzypenicillin 5 MU/ Vial Inj.	Highest priced generic				1			per vial.	
	Most sold generic				1			per vial	
	Lowest-priced generic				1			per vial	
Bisoprolol 5 mg cap/tab	Highest priced generic				60			per cap/tab	
	Most sold generic				60			per cap/tab	
	Lowest-priced generic				60			per cap/tab	
Captopril 25 mg cap/tab	Highest priced generic				100			per cap/tab	
	Most sold generic				100			per cap/tab	
	Lowest-priced generic				100			per cap/tab	
Carbamazepine 200 mg cap/tab	Highest priced generic				100			per cap/tab	
	Most sold generic				100			per cap/tab	
	Lowest-priced generic				100			per cap/tab	
Cefriaxone injection 1 g/vial vial	Highest priced generic				1			per vial	
	Most sold generic				1			per vial	

	Lowest-priced generic				1			per vial	
Cimetidine 400 mg cap/tab	Highest priced generic				100			per cap/tab	
	Most sold generic				100			per cap/tab	
	Lowest-priced generic				100			per cap/tab	
Ciprofloxacin 500 mg cap/tab	Highest priced generic				100			per cap/tab	
	Most sold generic				100			per cap/tab	
	Lowest-priced generic				100			per cap/tab	
Cotrimoxazole 80 + 400 mg cap/tab	Highest priced generic				1000			per cap/tab	
	Most sold generic				1000			per cap/tab	
	Lowest-priced generic				1000			per cap/tab	
Co-trimoxazole suspension 8+40 mg/ml mililitre	Highest priced generic				100			per mililitre	
	Most sold generic				100			per mililitre	
	Lowest-priced generic				100			per mililitre	
Diazepam 5 mg cap/tab	Highest priced generic				100			per cap/tab	
	Most sold generic				100			per cap/tab	
	Lowest-priced generic				100			per cap/tab	
Diazepam 5 mg/ ml Inj., 2 ml	Highest priced generic				2			per mililitre	
	Most sold generic				2			per mililitre	
	Lowest-priced generic				2			per mililitre	
Diclofenac 50 mg cap/tab	Highest priced generic				100			per cap/tab	
	Most sold generic				100			per cap/tab	
	Lowest-priced generic				100			per cap/tab	
Ergometrine Maleate 200 mg/ ml Inj., 1 ml	Highest priced generic				1			per mililitre	

	Most sold generic				1			per mililitre	
	Lowest-priced generic				1			per mililitre	
Erythromycin 250 mg cap/tab	Highest priced generic				1000			per cap/tab	
	Most sold generic				1000			per cap/tab	
	Lowest-priced generic				1000			per cap/tab	
Ethosuximide 250 mg cap/tab	Highest priced generic				100			per cap/tab	
	Most sold generic				100			per cap/tab	
	Lowest-priced generic				100			per cap/tab	
Fluconazole 200 mg cap/tab	Highest priced generic				100			per cap/tab	
	Most sold generic				100			per cap/tab	
	Lowest-priced generic				100			per cap/tab	
Gentamicin 40 mg/ ml Inj., 2 ml	Highest priced generic				2			per mililitre	
	Most sold generic				2			per mililitre	
	Lowest-priced generic				2			per mililitre	
Griseofulvin 125 mg cap/tab	Highest priced generic				100			per cap/tab	
	Most sold generic				100			per cap/tab	
	Lowest-priced generic				100			per cap/tab	
Hydrochlorothiazide 25 mg cap/tab	Highest priced generic				1000			per cap/tab	
	Most sold generic				1000			per cap/tab	
	Lowest-priced generic				1000			per cap/tab	
Ibuprofen 200 mg cap/tab	Highest priced generic				1000			per cap/tab	
	Most sold generic				1000			per cap/tab	
	Lowest-priced generic				1000			per cap/tab	

Insulin soluble 100 IU/ ml Inj., 10 ml Vial	Highest priced generic				10			per mililitre	
	Most sold generic				10			per mililitre	
	Lowest-priced generic				10			per mililitre	
Insulin Zink Susp. 100 IU/ ml Inj., 10 ml Vial	Highest priced generic				10			per mililitre	
	Most sold generic				10			per mililitre	
	Lowest-priced generic				10			per mililitre	
Magnesium sulphate 50 % , 2 ml	Highest priced generic				2			per mililitre	
	Most sold generic				2			per mililitre	
	Lowest-priced generic				2			per mililitre	
Mebendazole 500 mg cap/tab	Highest priced generic				100			per cap/tab	
	Most sold generic				100			per cap/tab	
	Lowest-priced generic				100			per cap/tab	
Metformin 500 mg cap/tab	Highest priced generic				90			per cap/tab	
	Most sold generic				90			per cap/tab	
	Lowest-priced generic				90			per cap/tab	
Methyldopa 250 mg cap/tab	Highest priced generic				100			per cap/tab	
	Most sold generic				100			per cap/tab	
	Lowest-priced generic				100			per cap/tab	
Metronidazole 200/ 250 mg cap/tab	Highest priced generic				1000			per cap/tab	
	Most sold generic				1000			per cap/tab	
	Lowest-priced generic				1000			per cap/tab	
Misoprostol 200 mg cap/tab	Highest priced generic				100			per cap/tab	
	Most sold generic				100			per cap/tab	

	Lowest-priced generic				100			per cap/tab	
Omeprazole 20 mg cap/tab	Highest priced generic				30			per cap/tab	
	Most sold generic				30			per cap/tab	
	Lowest-priced generic				30			per cap/tab	
Oxytocin 10 IU/ ml Inj., 1 ml	Highest priced generic				1			per mililitre	
	Most sold generic				1			per mililitre	
	Lowest-priced generic				1			per mililitre	
Paracetamol 500 mg cap/tab	Highest priced generic				1000			per cap/tab	
	Most sold generic				1000			per cap/tab	
	Lowest-priced generic				1000			per cap/tab	
Paracetamol suspension 24 mg/ml mililitre	Highest priced generic				100			per mililitre	
	Most sold generic				100			per mililitre	
	Lowest-priced generic				100			per mililitre	
Paraldehyd 10 ml/ vial Inj	Highest priced generic				10			per mililitre	
	Most sold generic				10			per mililitre	
	Lowest-priced generic				10			per mililitre	
Phenobarbital 200 mg/ ml Inj., 1 ml	Highest priced generic				1			per mililitre	
	Most sold generic				1			per mililitre	
	Lowest-priced generic				1			per mililitre	
Phenobarbital tab 30 mg cap/tab	Highest priced generic				1000			per cap/tab	
	Most sold generic				1000			per cap/tab	
	Lowest-priced generic				1000			per cap/tab	
Phenytoin Na 50 mg/ ml Inj.	Highest priced generic				1			per mililitre	

	Most sold generic				1			per mililitre	
	Lowest-priced generic				1			per mililitre	
Phenytoin Na tab 100 mg cap/tab	Highest priced generic				100			per cap/tab	
	Most sold generic				100			per cap/tab	
	Lowest-priced generic				100			per cap/tab	
Praziquantel 600 mg cap/tab	Highest priced generic				100			per cap/tab	
	Most sold generic				100			per cap/tab	
	Lowest-priced generic				100			per cap/tab	
Salbutamol inhaler 100 mcg/dose	Highest priced generic				200			per dose	
	Most sold generic				200			per dose	
	Lowest-priced generic				200			per dose	
Simvastatin 20mg cap/tab	Highest priced generic				30			per cap/tab	
	Most sold generic				30			per cap/tab	
	Lowest-priced generic				30			per cap/tab	
Sodium Chloride 0,9 % Infusion, 1 l	Highest priced generic				1000			per mililitre	
	Most sold generic				1000			per mililitre	
	Lowest-priced generic				1000			per mililitre	
Sodium Valproate 200 mg cap/tab	Highest priced generic				100			per cap/tab	
	Most sold generic				100			per cap/tab	
	Lowest-priced generic				100			per cap/tab	
Tetracycline 1 % m/m eye ointment	Highest priced generic				3.5			per eye ointment	
	Most sold generic				3.5			per eye ointment	
	Lowest-priced generic				3.5			per eye ointment	

Zink sulfate 20 mg cap/tab	Highest priced generic				100			per cap/tab	
	Most sold generic				100			per cap/tab	
	Lowest-priced generic				100			per cap/tab	

Before leaving the facility :

Data collectors should check that the data collection form is legible, accurate and complete before leaving the facility and returning completed forms to the area supervisor. They should report any problems as soon as possible. They should also check to see whether at least half of the survey medicines were available, to determine whether a visit to a back-up facility is required.
