# Supplementary material for: The availability, prices and affordability of essential medicines in Malawi: A cross-sectional study
Source: PLoS One. 2019 Feb 12;14(2):e0212125. doi: 10.1371/journal.pone.0212125 (PMC6372227; doi:10.1371/journal.pone.0212125)
Supplement: S4 Table — (PDF) [file pone.0212125.s004.pdf]

S4 Table. Calculation of medicine treatment costs and affordability.

Medicine name	Strength	Course of treatment	Number of units per treatment	Retail pharmacies			CHAM facilities			Private clinics		
				Median price in US Cents	Price per course of treatment in US$	Number of days to afford treatment	Median price in US cents	Price per course of treatment in US$	Number of days to afford treatment	Median price in US cents	Price per course of treatment in US$	Number of days to afford treatment
Amitriptyline	25mg	3 tab/day, 30 days	90	2.07	1.86	1.40	1.86	1.67	1.26	3.44	3.10	2.34
Amoxicillin	250mg	6 cap/day, 7 days	42	3.44	1.45	1.09	4.13	1.74	1.31	4.13	1.74	1.31
Amoxicillin susp	25mg/mL	15 mL/day, 7 days	100	1.10	1.10	0.83	0.83	0.83	0.62	0.96	0.96	0.73
Benzylpenicillin inj	5MU/vial	4 vials/day, 3 days	12	86.12	10.33	7.80	74.06	8.89	6.70	112.30	13.48	10.17
Bisoprolol	5mg	2 tab/day, 30 days	60	13.53	8.12	6.13				10.28	6.17	4.66
Captopril	25mg	3 tab/day, 30 days	90	4.13	3.72	2.81	2.41	2.17	1.64	7.87	7.09	5.35
Carbamazepin	200mg	4 tab/day, 30 days	120	5.10	6.12	4.62	4.13	4.96	3.74	5.81	6.97	5.26
Ceftriaxone inj	1 g/vial	4 vials/day, 3 days	12	122.63	14.72	11.10	117.12	14.05	10.60	114.02	13.68	10.32
Ciprofloxacin	500mg	2 tab/day, 7 days	14	10.33	1.45	1.09	6.89	0.96	0.73	8.27	1.16	0.87
Cotrimoxazole	480mg	4 tab/day, 7 days	28	2.48	0.69	0.52	2.76	0.77	0.58	3.10	0.87	0.65
Cotrimoxazole susp	8+40 mg/mL	10 mL/day, 7 days	100	1.10	1.10	0.83	0.86	0.86	0.65	0.82	0.82	0.62
Diazepam	5mg	6 tab/day, 7 days	42	1.23	0.52	0.39	1.72	0.72	0.55	2.76	1.16	0.87
Diazepam inj	5mg/mL	6 amp/day, 2 days	12			-	35.82	4.30	3.24	69.79	8.37	6.32
Erythromycin	250mg	8 tab/day, 7 days	56	6.89	3.86	2.91	6.20	3.47	2.62	7.58	4.24	3.20
Fluconazole	200mg	1 tab/day, 30 days	30	21.36	6.41	4.83	27.56	8.27	6.24	33.99	10.20	7.69

				Retail pharmacies			CHAM facilities			Private clinics		
**Medicine name**	**Strength**	**Course of treatment**	**Number of units per treatment**	**Median price in US Cents**	**Price per course of treatment in US$**	**Number of days to afford treatment**	**Median price in US cents**	**Price per course of treatment in US$**	**Number of days to afford treatment**	**Median price in US cents**	**Price per course of treatment in US$**	**Number of days to afford treatment**
Gentamicin	40mg/mL	6 mL at once	6	13.78	0.83	0.62	20.67	1.24	0.94	37.32	2.24	1.69
Hydrochlorothiazide	25mg	1 tab/day, 30 days	30	0.92	0.28	0.21	1.38	0.41	0.31	3.10	0.93	0.70
Insulin soluble inj	100IU/mL, 10mL	30 IU/day, 30 days	10	206.68	20.67	15.59	107.47	10.75	8.11	130.90	13.09	9.88
Insulin zinc susp inj	100IU/mL, 10mL	30 IU/day, 30 days	10			-	168.79	16.88	12.73	130.90	13.09	9.88
Magnesium sulphate 50 % inj	50%, 2mL	10 amp/ course	10			-	37.20	3.72	2.81	94.38	9.44	7.12
Metformin	500mg	4 tab/day, 30 days	120	3.44	4.13	3.12	2.76	3.31	2.49	5.51	6.61	4.99
Methyldopa	250mg	6 tab/day, 30 days	180	7.23	13.02	9.82	5.51	9.92	7.48	5.74	10.33	7.80
Metronidazol	200/250mg	6 tab/day, 7 days	42	1.84	0.77	0.58	2.76	1.16	0.87	2.66	1.12	0.84
Misoprostol	200mcg	4 tab at once	4	41.34	1.65	1.25	22.39	0.90	0.68	89.56	3.58	2.70
Oxytocin	10IU/mL, 1mL	2 amp at once	2			-	55.11	1.10	0.83	12.50	2.50	0.62
Phenobarbital sodium inj	200mg/mL, 1mL	3 amp/day, 3 days	9			-	71.65	6.45	4.86	0.96	0.96	9.06
Phenobarbital sodium	30mg	3 tab/day, 30 days	90	1.38	1.24	0.94	1.38	1.24	0.94	112.30	13.48	1.54
Phenytoin sodium	100mg	3 tab/day, 30 days	90	8.61	7.75	5.85	0.69	0.62	0.47	7.87	7.09	-
Salbutamol inhaler	100 mcg/dose	1 tube of 200 doses/30 days	200	2.07	4.13	3.12	1.38	2.76	2.08	114.02	13.68	3.12
Simvastatin	20mg	1 tab/day, 30 days	30	13.53	4.06	3.06			-	3.54	0.99	-
Sodium chloride inj	0.9%, 1Litre	2L/day, 3 days	6000			-	0.24	14.47	10.91	8.27	1.16	15.61

				Retail pharmacies			CHAM facilities			Private clinics		
**Medicine name**	**Strength**	**Course of treatment**	**Number of units per treatment**	**Median price in US Cents**	**Price per course of treatment in US$**	**Number of days to afford treatment**	**Median price in US cents**	**Price per course of treatment in US$**	**Number of days to afford treatment**	**Median price in US cents**	**Price per course of treatment in US$**	**Number of days to afford treatment**
Sodium valproate	200mg	4 tab/day, 30 days	120	13.09	15.71	11.85	6.89	8.27	6.24	3.10	0.87	47.23
